# 574 Want some s’more? The hidden risks of marshmallows

**DOI:** 10.1093/jbcr/irac012.202

**Published:** 2022-03-23

**Authors:** Francesca Ghini, Mehul Thakkar, Timothy Schrire, Bartlomiej Bednarz

**Affiliations:** North Bristol NHS Trust, Bristol, England; Glasgow Royal Infirmary, London, England; North Bristol NHS Trust, Bristol, England; North Bristol NHS Trust, Bristol, England

## Abstract

**Introduction:**

Marshmallows are a spongy confectionery commonly made with gum Arabic or gelatine, corn syrup, sugar, and flavouring. It is common, in many Anglophone countries, to toast marshmallows as an outdoor childhood pastime. Being an aerated aqueous mixture, marshmallows have unique properties when exposed to high temperatures, resulting in a Maillard reaction between the sugars and proteins. They therefore present a distinctive and significant method of burn injury.

**Methods:**

We reviewed our regional online burns database for marshmallow related burns in the last six years to collate data on patient demographics, burn size and depth, method of injury, and outcome.

**Results:**

Between 2015-2020, our Regional Burns Centre treated 35 patients with an average age of 8.3 years (range 4-14). The most commonly injured area was the face in 44% of the cases (lips specifically in 25%), followed by hands in 36%. The burn depth extended from deep dermal to full thickness in 6% of cases and the total body surface area affected was 0.3% on average. No admission or surgical intervention was required. A significant proportion of burns were associated with camping, open fires or barbecues (63%), however the recorded mechanisms of injury were not limited to these activities alone. 77% of injuries occurred between Friday and Sunday.

**Conclusions:**

Marshmallow burns are hazardous due to their composition and response to heat. The adhesive nature of a toasted marshmallow leads to a deeper pattern of burn injury, even though, the majority can usually be managed conservatively due to the limited area affected.

